# A Chemical-Induced, Seed-Soaking Activation Procedure for Regulated Gene Expression in Rice

**DOI:** 10.3389/fpls.2017.01447

**Published:** 2017-08-21

**Authors:** Zaijie Chen, Qianqian Cheng, Chanquan Hu, Xinrui Guo, Ziqiang Chen, Yan Lin, Taijiao Hu, Maria Bellizzi, Guodong Lu, Guo-Liang Wang, Zonghua Wang, Songbiao Chen, Feng Wang

**Affiliations:** ^1^Fujian-Taiwan Joint Center for Ecological Control of Crop Pests, Fujian Agriculture and Forestry University Fuzhou, China; ^2^Biotechnology Research Institute, Fujian Academy of Agricultural Sciences Fuzhou, China; ^3^Department of Plant Pathology, The Ohio State University, Columbus OH, United States

**Keywords:** *Oryza sativa*, regulated gene expression, XVE system, DNA recombination, seed-soaking

## Abstract

Inducible gene expression has emerged as a powerful tool for plant functional genomics. The estrogen receptor-based, chemical-inducible system XVE has been used in many plant species, but the limited systemic movement of inducer β-estradiol in transgenic rice plants has prohibited a wide use of the XVE system in this important food crop. Here, we constructed an improved chemical-regulated, site-specific recombination system by employing the XVE transactivator in combination with a Cre/*loxP*-FRT system, and optimized a seed-soaking procedure for XVE induction in rice. By using a *gus* gene and an hpRNAi cassette targeted for *OsPDS* as reporters, we demonstrated that soaking transgenic seeds with estradiol solution could induce highly efficient site-specific recombination in germinating embryos, resulting in constitutive and high-level expression of target gene or RNAi cassette in intact rice plants from induced seeds. The strategy reported here thereby provides a useful gene activation approach for effectively regulating gene expression in rice.

## Introduction

Inducible gene expression systems are powerful tools for basic research in plant functional genomics, and for plant biotechnology applications such as the deletion of selectable marker genes in transgenic plants (Moore et al., 2006). Over the last two decades, heat-inducible system (Zhang et al., 2003; Khattri et al., 2011; Nandy and Srivastava, 2012), and chemical-inducible systems responding to various inducers, such as glucocorticoid (Schena et al., 1991; Aoyama and Chua, 1997), copper (Mett et al., 1993; McKenzie et al., 1998), tetracycline (Weinmann et al., 1994; Bohner et al., 1999), herbicide safeners (De Veylder et al., 1997), ethanol (Caddick et al., 1998; Salter et al., 1998), ecdysone (Martinez et al., 1999), and β-estradiol (Bruce et al., 2000; Zuo et al., 2000) have been developed. Compared to constitutive gene expression driven by constitutive promoters, conditional gene expression controlled by the chemical-inducible systems has provided a more flexible approach for functional dissection of developmentally important genes, especially if the genes related to regeneration, growth, reproduction, or lethality.

Among the developed chemical-inducible systems, the estrogen receptor-based transactivator XVE (Zuo et al., 2000) appears to be one of the most reliable and efficient systems. XVE consists of a DNA-binding domain of the bacterial repressor LexA, a transactivating domain of herpes simplex virus VP16, and a human estrogen receptor. Characterization of the XVE system in *Arabidopsis* demonstrated that the target gene expression controlled by this system was highly induced and tightly regulated upon estradiol induction (Zuo et al., 2000). The XVE system has been applied to different plant species, including basal land plant moss (Kubo et al., 2013), flowering dicot (Zuo et al., 2000, 2001; Guo et al., 2003; Zhang et al., 2007; Schlücking et al., 2013) and monocot (Sreekala et al., 2005; Okuzaki et al., 2011) plants.

Rice (*Oryza sativa* L.) is one of the world’s most important staple food crops and has been established as a model organism for the functional genomics of monocot plants. The availability of highly accurate genomic sequences of both *indica* (Yu et al., 2002) and *japonica* (International Rice Genome Sequencing Project, 2005) subspecies, and many other accumulated genomic and molecular resources (Kikuchi et al., 2003; Jung et al., 2008), has greatly promoted genomic and genetic studies in rice. The XVE system has been tested in rice calli to produce marker-free transgenic plants (Sreekala et al., 2005), and in both calli and seedlings to investigate target gene expression patterns (Okuzaki et al., 2011). The two reports showed that the XVE system is functional in regulating gene expression in rice calli and roots. In the leaves of intact plantlets treated with estradiol through root absorption, however, target gene induction was observed at a very low level (Okuzaki et al., 2011), becoming one of the major limitations for this system to be widely used in discovering gene function in rice.

In the present study, based on detailed investigation of XVE-controlled gene expression patterns in calli, detached leaf pieces, and intact plantlets, we proposed that limited systemic movement of estradiol might be the key reason for low inducibility in intact rice plants. To meet the need for high-level target gene expression in intact rice plants, we further constructed an improved XVE-controlled, site-specific recombination system and optimized the induction procedure for rice seeds. Experiments based on a reporter *gus* gene and an hpRNAi cassette targeted to a rice phytoene desaturase (*OsPDS*) gene showed that soaking transgenic seeds with estradiol solution could activate highly efficient site-specific recombination in germinating embryos, resulting in constitutive activation of the target gene or RNAi cassette in intact rice plants. Our method thereby provides a useful gene activation approach for functional genomics in rice.

## Materials and Methods

### Plasmid Construction

Plant expression vectors pUX-GUS, pXCL-GUS, pXCLF-GUS, and pXCLF-PDSi were constructed following standard molecular manipulation procedures (Sambrook and Russell, 2001), except that the TOPO cloning and the LR clonase reaction for constructing pXCLF-PDSi were carried out by following Gateway cloning protocol (Invitrogen, Carlsbad, CA, United States). All plasmids derived from PCR products or synthetic oligonucleotides were verified by sequencing. Details of plasmid construction are available in Supplementary Table S1.

### Rice Protoplast Transient Expression Assay

Protoplasts were isolated from 2-week-old etiolated seedlings of rice, *O. sativa* cv. Nipponbare. PEG-mediated protoplast transfection was carried out by following previously described procedures (Chen et al., 2006). After transfection, estradiol was applied to protoplasts immediately with final concentration of 20 μM. Protoplasts were harvested after incubation for 12, 24, 36, and 48 h, respectively, and subjected to GUS assay.

### Transformation of Rice Calli and Production of Transgenic Rice

Plant expression constructs pUX-GUS, pXCL-GUS, pXCLF-GUS, and pXCLF-PDSi were introduced into the *Agrobacterium tumefaciens* strain LBA4404. Rice callus was induced from the embryos of mature seeds of cv Nipponbare. *Agrobacterium*-mediated transformation of rice calli was conducted using the method described previously (Hiei et al., 1994). The hygromycin-resistant transgenic calli were subjected to estradiol induction assays or transferred to regeneration medium. Regenerated transgenic plants and their progeny were grown in a greenhouse. Transgenic lines contain a single copy of T-DNA insertion based on genetic segregation analysis of T_1_ generation, Southern blotting, and quantitative real-time PCR (qPCR) analysis of transgene copy number were used for further analyses (Supplementary Figure S1).

### Estradiol Treatment

17-β-estradiol (Sigma–Aldrich, St. Louis, MO, United States) was dissolved to 20 mM in dimethyl sulfoxide (DMSO), and stored at -20°C as stock solution. The stock solution was added to sterilized water, or solid medium at a final concentration of 20 μM immediately before use. The same volume of DMSO was added to the negative control solution or medium without estradiol. For induction treatment, rice transgenic calli were directly cultured on N6 medium supplemented with estradiol, and rice detached leaf pieces or rice seeds were submerged in estradiol water solution. In whole-plantlet treatment, seedlings germinated on 1/2 MS medium plates were transferred to small tubes or flasks, and plantlet roots were submerged in estradiol water solution.

### GUS Assays

GUS assays were performed as described by Jefferson et al. (1987). The fluorometric assay was carried out using 4-methylumbelliferyl-β-D-glucuronide (MUG; Sigma–Aldrich, St. Louis, MO, United States) as substrate, and the histochemical staining was performed using X-Gluc (Sigma–Aldrich, St. Louis, MO, United States) as substrate.

### PCR, DNA Sequencing, qPCR, and qRT-PCR Analyses

Genomic DNA was extracted from rice tissues by using the CTAB method as described by Murray and Thompson (1980). PCR for investigating the recombination events in induced rice plants was carried out under standard conditions. The PCR products from primers LF-P1/LF-P4 or LF-P1/LF-P5 amplified from the induced LF-GUS or LF-PDSi plants were sequenced. The sequencing results were analyzed using Sequence Scanner software (Applied Biosystems). Real-time qPCR was performed to determine transgene copy number in homozygous lines by following previously described method (Wang et al., 2015). The single-copy rice endogenous gene *RBE4* (Wang et al., 2015) was used as a reference and the *Hpt* gene was used as probe.

Total RNA was isolated from rice leaf tissues by using TRIzol reagent (Invitrogen Life Technologies, Carlsbad, CA, United States) and was subsequently treated with RNase-free DNase I (Takara, Dalian, China) to remove DNA contamination. Reverse transcription was carried out using 2.5 μg of DNase I-treated total RNA with the RevertAid First Strand cDNA Synthesis Kit (Thermo Scientific, Lithuania, EU) according to the manufacturer’s instructions. The qPCR was performed on an ABI 7500 real-time PCR system (Applied Biosystems, Carlsbad, CA, United States) using SYBR Premix Ex Taq (Takara, Dalian, China) according to the manufacturer’s instructions.

All primers used for PCR, qPCR, and qRT-PCR were listed in the Supplementary Table S2.

### Southern Blot Analysis

Southern blot analysis was performed following standard procedures (Sambrook and Russell, 2001). Rice genomic DNA (about 10 μg) was digested with appropriate restriction enzymes. The digested DNA was separated on a 0.8% agarose gel, and was transferred onto a Hybond-N+ nylon membrane (Amersham Biosciences, Buckinghamshire, United Kingdom). The blot was hybridized using a 3′-end fragment of the maize ubiquitin-1 promoter labeled by Alkphos Direct Labelling Reagents (Amersham Bioscience).

## Results

### XVE Stringently and Efficiently Regulates Gene Expression in Rice Cells and Tissues upon Direct Contact with Estradiol

**FIGURE 1:**
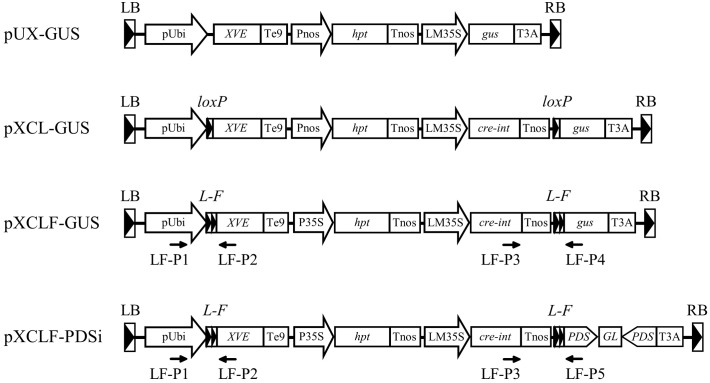
Schematic diagrams of the T-DNA region of vectors used in this study. pUbi, maize ubiquitin-1 promoter; *XVE*, chimeric transactivator containing the regulator domain of an estrogen receptor (Zuo et al., 2000); Te9, rbcS E9 terminator; Pnos, nopaline synthase promoter; *hpt*, hygromycin phosphotransferase gene; Tnos, nopaline synthase terminator; LM35S, 8 × LexA DNA binding site fused with the -46 CaMV 35S minimal promoter; *gus*, β-glucuronidase gene; *cre-int*, bacteriophage P1 Cre recombinase gene modified with an intron; T3A, rbcS 3A terminator; P35S, CaMV 35S promoter; *PDS*, a fragment of the rice phytoene desaturase gene; *GL*, *gus* linker fragment; *loxP*, specific recognition site of Cre; *L-F*, fusion sequence of *loxP* and *FRT*; LB, T-DNA left border; RB, T-DNA right border; LF-P1–LF-P5, primers used for PCR analysis.

**FIGURE 2:**
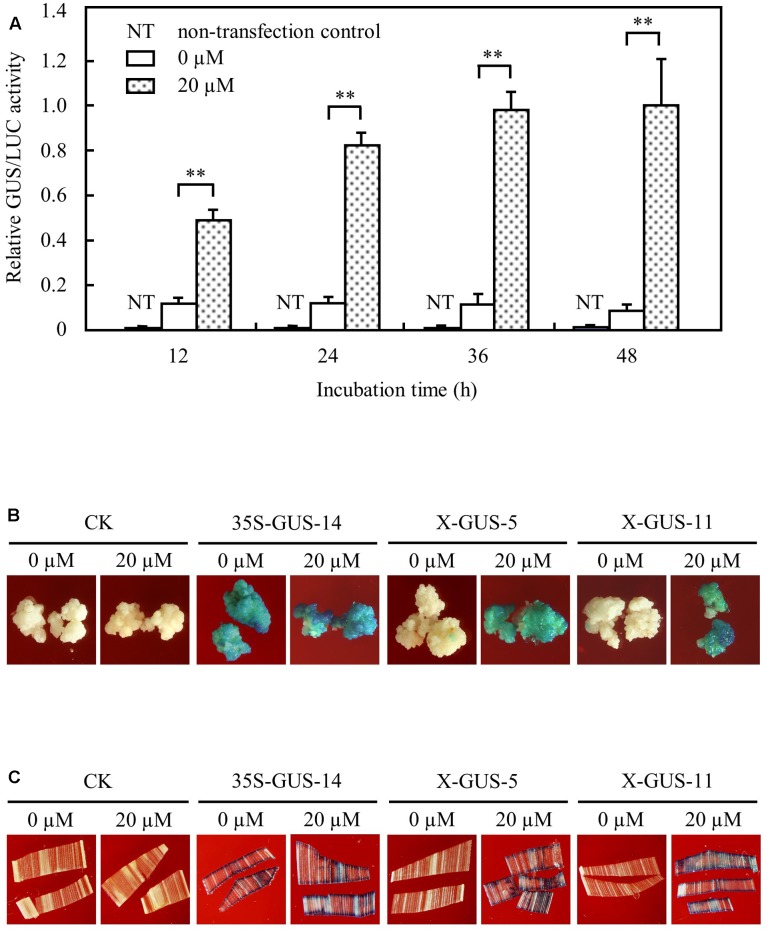
XVE-controlled GUS expression in rice protoplast, calli, and leaf tissues upon direct contact with estradiol. **(A)** Transient expression assay of XVE-controlled GUS expression in rice protoplasts. Protoplasts transfected with construct pUX-GUS were treated with 0 or 20 μM estradiol for 12, 24, 36, and 48 h, respectively. GUS expressions are represented as relative GUS/LUC activity. ^∗∗^*P* < 0.01, *t*-test. **(B)** GUS staining of rice calli induced with 0 or 20 μM estradiol. CK, non-transgenic wild type control; 35S-GUS-14, line #14 of rice calli transformed with construct pCAMBIA1305, in which the *gus* gene was driven by a 35S promoter; X-GUS-5 and X-GUS-11, line #5 and #11 of rice calli transformed with construct pUX-GUS. **(C)** GUS staining of detached transgenic leaf pieces induced with 0 or 20 μM estradiol.

We generated the estrogen-inducible expression construct *pUX-GUS* (**Figure 1**) using a *gus* gene as a reporter, because the GUS system has been proved to be a reliable and extremely sensitive reporter allowing both quantitative and histochemical assessment of gene activity in plants (Jefferson et al., 1987). The *pUX-GUS* construct was first tested transiently in rice protoplasts. The transfected rice protoplasts were treated with 20 μM estradiol. Transient expression assays showed that the GUS expression level in transfected rice protoplasts was significantly induced after 12 h induction, and the induced level was increased gradually after 24, 36, and 48 h inductions (**Figure 2A**). In transfected rice protoplasts without treatment with estradiol, GUS expression was detected at a very low level after 24 h incubation, and remained at a similar low level after 36 or 48 h incubation (**Figure 2A**).

To further analyze XVE-controlled gene expression in stably integrated transgenic rice tissues, pUX-GUS and a control construct pCAMBIA1305.2^1^ in which a *gus* gene is driven by a constitutive CaMV 35S promoter (Odell et al., 1985), were then used for stable transformation of rice calli. Hygromycin-resistant transgenic calli after selective screening were divided into two groups and were cultured on N6 medium with or without estradiol for induction analysis. Histochemical staining detected strong GUS expression in pUX-GUS-transformed calli (named X-GUS) induced with 20 μM estradiol for 3 days, similar to that of calli transformed with pCAMBIA1305.2 (named 35S-GUS) (**Figure 2B**). By contrast, almost no GUS staining was observed in X-GUS calli without treatment with estradiol during the culture period (**Figure 2B**).

Transgenic calli were further cultured to regenerate rice plants, and a total of 17 independent T_0_ X-GUS lines were obtained. Small pieces of transgenic plant leaves were cut and exposed to 20 μM estradiol for 24 h, and the GUS activity was determined by histochemical staining. From 17 independent lines, two lines showed no GUS staining in detached leaf pieces either treated or not treated with estradiol, and one line showed weak GUS staining in both induced and uninduced leaf pieces. For the remaining 14 lines, while no GUS staining was detected in leaf pieces without treatment, clear GUS staining was observed in estradiol treated-leaf pieces (**Figure 2C**). Taken together, these results demonstrated that the XVE system could stringently and efficiently regulate gene expression in rice cells and tissues.

### XVE Mediates Low Level Inducibility of Target Gene in the Leaves of Intact Plants Induced with Estradiol through Root Absorption

The transgenic X-GUS, and 35S-GUS rice plants were grown to produce progeny of advanced generation for further analysis. Among the 14 screened X-GUS lines, #5 and #11 were examined to confer a strong estradiol-inducible GUS expression pattern in detached leaf pieces, and contain a single copy of T-DNA insertion based on genetic segregation analysis of T_1_ generation, Southern blotting and qPCR analysis of transgene copy number (Supplementary Figure S1). Detached leaf pieces of X-GUS plants of each different generation (T_1_ to T_4_) were treated with estradiol, and GUS staining could be observed in induced leaf tissues but not in uninduced tissues, similar to that of T_0_ generation plants (**Figure 2C**). These results suggested that XVE-controlled GUS expression pattern could be inherited stably over generations.

**FIGURE 3:**
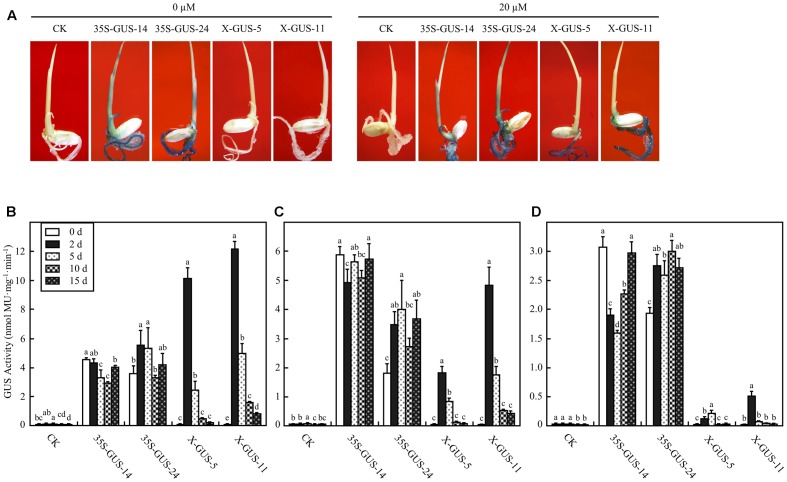
XVE-controlled GUS expression in intact transgenic rice seedlings treated with estradiol through root absorption. **(A)** GUS staining of rice plantlets exposed to 0 or 20 μM estradiol via root immersion for 2 days. CK, non-transgenic wild type control; 35S-GUS-14 and 35S-GUS-24, pCAMBIA1305-transformed rice line #14 and #24; X-GUS-5 and X-GUS-11, pUX-GUS-transformed rice line #5 and #11. **(B–D)** GUS fluorometric assay of the roots **(B)**, stems **(C)**, and leaves **(D)** of rice plantlets cultured in 0 or 20 μM estradiol via root immersion. Three-leaf-old seedlings of rice plants were cultured in liquid solution, and the roots, shoots, or leaves of the cultured seedlings were collected for GUS assay on days 0, 2, 5, 10, and 15, respectively. Different letters indicate groups with significant differences (ANOVA, *p* < 0.05).

To examine the estrogen-induced target gene expression patterns in intact rice plantlets, 4-day-old seedlings of T_5_ homozygous lines were cultured in liquid solution *via* a root absorption manner for 2 days. The cultured seedlings were subjected to histochemical staining. While no GUS expression was observed in plantlets of X-GUS-#5 or X-GUS-#11 cultured in liquid medium without estradiol, strong GUS staining was observed in the roots of X-GUS-#5 and X-GUS-#11 cultured in estradiol solution (**Figure 3A**). However, when compared with 35S-GUS plants, X-GUS plantlets showed only weak GUS staining in leaf tissues (**Figure 3A**). A fluorometric assay was also performed to detect GUS expression level in transgenic plantlets. Three-leaf-stage seedlings of transgenic plants were cultured in liquid solution for different induction periods (0, 2, 5, 10, and 15 days), and the roots, shoots, and leaves of the cultured seedlings were collected and evaluated for GUS activity. With 20 μM estradiol induction for 2 days, GUS expression was induced in X-GUS plantlets. In the roots of X-GUS-#5 and X-GUS-#11, GUS level was about two times higher than or similar to that of 35S-GUS plants (**Figures 3B,C**). However, GUS level in the leaves of X-GUS-#5 and X-GUS-#11 was much lower than that of 35S-GUS plants (**Figure 3D**), similar to previous report that XVE-controlled GFP was expressed at a low level in the leaves of transgenic seedlings cultured on the inductive medium (Okuzaki et al., 2011). Fluorometric assays also revealed that GUS expression level in X-GUS plantlets was gradually decreased after induction for 5, 10, and 15 days, respectively (**Figures 3B–D**).

### Construction of an Improved Chemical-Regulated, Site-Specific Recombination System for Rice

Unlike *Arabidopsis*, rice plants are larger in size, and have a relatively longer life cycle. The difficulty in inducing target genes to be expressed at a high level in the leaves of intact plantlets, and the relatively short induction duration, have limited the use of the XVE system in rice. We reasoned that a system permitting constitutive gene expression after chemical induction would be more feasible for rice. We thus designed an alternative approach by employing the XVE system coupled with a site-specific recombination cassette. A basic construct pXCL-GUS, in which a XVE-controlled Cre/*loxP*-mediated recombination would lead to fusion of a maize (*Zea mays*) ubiquitin-1 promoter (Christensen et al., 1992) to the *gus* gene upon estradiol induction (**Figures 1**, **6D**), was developed initially. However, rice transformation experiments showed that pXCL-GUS gave only very low transformation efficiency (Supplementary Table S3). A modified construct pXCLF-GUS (**Figure 1**), in which the original *nos* promoter for the selectable marker gene *hpt* was replaced with a CaMV 35S promoter, was constructed. In pXCLF-GUS, the two directly oriented *loxP* sequences were also replaced with two *loxP-FRT* fusion sequences which were previously reported to have enhanced excision efficiency mediated by Cre recombinase (Luo et al., 2007). Rice transformation experiments showed that the modified pXCLF-GUS yielded a transformation efficiency about 15 times higher than that of pXCL-GUS (Supplementary Table S3).

### The XVE-controlled Cre/*loxP-FRT* (XCLF) System Permits Inducible and High-Level Expression of Target Genes in Transgenic Rice Plants

**FIGURE 4:**
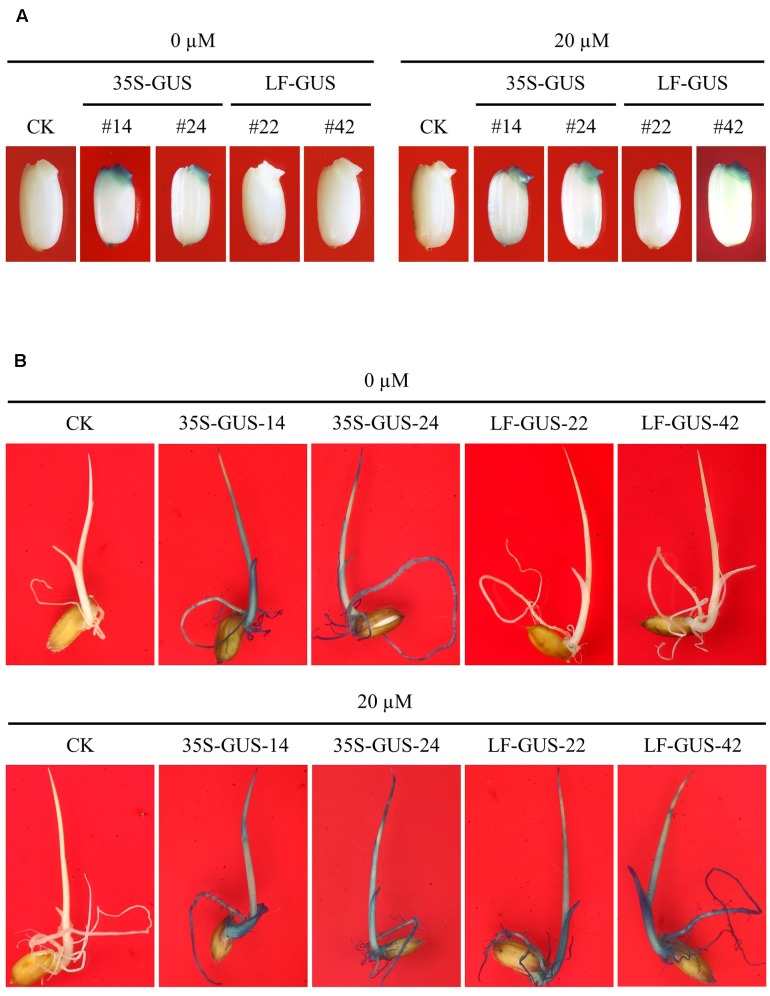
Induced GUS expression in germinating embryos and seedlings of LF-GUS transgenic rice. **(A)** GUS staining of rice seeds soaked in 0 or 20 μM estradiol for 3 days. CK, non-transgenic wild type control; 35S-GUS-14 and 35S-GUS-24, pCAMBIA1305-transformed rice line #14 and #24; LF-GUS-22 and LF-GUS-42, pXCLF-GUS-transformed rice line #22 and #42. **(B)** GUS staining of seedlings developed from seeds soaked and germinated in liquid solution with 0 or 20 μM estradiol.

The uninduced rice calli transformed with pXCLF-GUS (named LF-GUS) were cultured on media for regeneration, and a total of 18 independent T_0_ plants were obtained. T_0_ plants were transferred to soil and maintained in a greenhouse to produce T_1_ seeds. To induce expression of Cre recombinase, we soaked transgenic rice seeds in liquid solution instead of germinating seeds on solid medium. A 3-day soaking of seeds was observed to have a good effect on seed induction (**Figure 4A**). By histochemical staining assay, about 12 T_1_ independent lines were detected to display induced GUS expression upon seed-soaking treatment. In LF-GUS lines, GUS expression was detected in the embryos of LF-GUS transgenic seeds soaked in the inductive solution, but not in seeds soaked in the solution without estradiol (**Figure 4A**). The LF-GUS seedlings developed from induced seeds displayed a constitutive expression pattern of GUS, whereas no GUS or only very faint GUS spots were observed in uninduced LF-GUS seedlings (**Figure 4B**).

**FIGURE 5:**
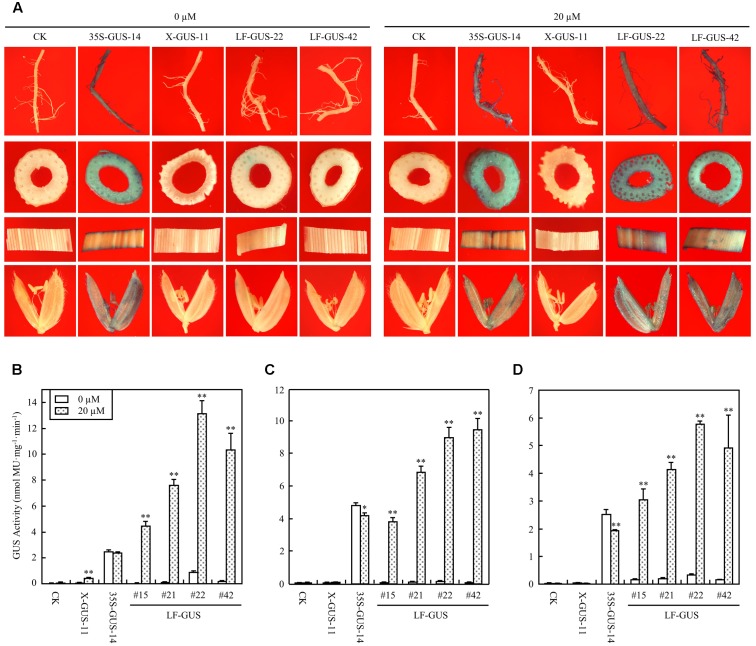
Induced GUS expression in mature LF-GUS transgenic rice plants. **(A)** GUS staining of the roots, stems, leaves, and florets of mature plants developed from seeds soaked in liquid solution with 0 or 20 μM estradiol. CK, non-transgenic wild type control; 35S-GUS-14, pCAMBIA1305-transformed rice line #14; X-GUS-11, pUX-GUS-transformed rice line #11; LF-GUS-22 and LF-GUS-42, pXCLF-GUS-transformed rice line #22 and #42. **(B–D)** GUS fluorometric assay of the roots **(B)**, stems **(C)** and leaves **(D)** of mature rice plants developed from seeds soaked and germinated in liquid solution with 0 or 20 μM estradiol. CK, non-transgenic wild type control; X-GUS-11, pUX-GUS-transformed rice line #11; 35S-GUS-14, pCAMBIA1305-transformed rice line #14; LF-GUS #15, #21, #22, and #42, pXCLF-GUS-transformed rice line #15, #21, #22, and #42. Statistical significances (^∗^*P* < 0.05 or ^∗∗^*P* < 0.01) between plants developed from seeds soaked in 0 or 20 μM estradiol were analyzed by Student’s *t*-test.

The T_2_ generation of four LF-GUS homozygous lines, #15, #21, #22, and #42 were subjected to more detailed analysis. In mature flowering plants developed from estradiol-induced LF-GUS seeds, constitutive GUS expression was detected in root, stem, leaf, and floret tissues of LF-GUS plants by histochemical staining (**Figure 5A**). Consistent with results observed in T_1_ seedlings, almost no GUS staining or only very faint GUS spots were observed in tissues of LF-GUS plants developed from uninduced seeds (**Figure 5A**). Fluorometric assays confirmed that GUS was highly expressed in all tested root, stem, and leaf tissues in the induced mature LF-GUS plants (**Figures 5B–D**). While the level of GUS activity in root, stem, or leaf tissues of LF-GUS-#15 plants were comparable to that of 35S-GUS-#14 plants, GUS expression level of LF-GUS-#21, #22, or #42 plants was about 1.5–2 times higher than that of 35S-GUS-#14 plants (**Figures 5B–D**). In uninduced LF-GUS plants, only a low GUS level similar to that of non-transgenic plants was detected (**Figures 5B–D**). No GUS expression was detected in tissues of mature plants of X-GUS line #11 developed from seeds germinated in liquid solution with or without inducer (**Figures 5A–D**), indicating that estradiol induction in seeds did not persist to later times.

**FIGURE 6:**
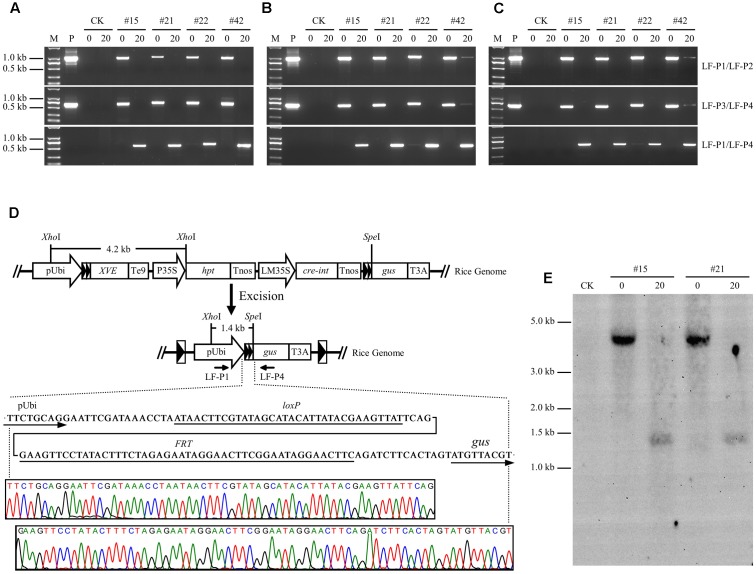
Molecular characterization of chemical-induced site-specific DNA recombination in LF-GUS transgenic rice plants. **(A–C)** PCR analysis of genomic DNA extracted from the roots **(A)**, stems **(B)**, and leaves **(C)** of LF-GUS plants. M, DNA molecular marker; P, plasmid control pXCLF-GUS; CK, non-transgenic wild type control; #15, #21, #22, and #42, pXCLF-GUS-transformed rice line #15, #21, #22, and #42; 0, plants developed from seeds soaked and germinated in liquid solution with 0 μM estradiol; 20, plants developed from seeds soaked and germinated in liquid solution with 20 μM estradiol. **(D)** Sequence confirmation of site-specific DNA recombination in the genome of induced LF-GUS plants. The diagram represents the putative reconstituted structure after recombination between two *loxP-FRT* sites. Sequence below the diagram indicates the predicted nucleotide sequence of the junction regions at the recombined *loxP-FRT* site. The bottom chromatogram shows sequencing results of the LF-P1/LF-P2 amplified product from induced LF-GUS plants. **(E)** Southern blot analysis of genomic DNA extracted from leaves of LF-GUS plants. CK, non-transgenic wild type control; #15 and #21, pXCLF-GUS-transformed rice line #15 and #21; 0, plants developed from seeds soaked and germinated in liquid solution with 0 μM estradiol; 20, plants developed from seeds soaked and germinated in liquid solution with 20 μM estradiol. Rice genomic DNA was digested with *Xho*I and *Spe*I. The blot was hybridized with a 3′-end fragment of the maize ubiquitin-1 promoter.

PCR analysis was performed on genomic DNA isolated from root, stem, and leaf tissues to investigate the recombination events in LF-GUS plants. Using specific primers LF-P1/LF-P2 targeted to the joint region of *pUbi-loxP-FRT-XVE*, and LF-P3/LF-P4 to the joint region of *Cre-int-Tnos-loxP-FRT-gus* of the non-recombinant T-DNA (**Figure 1**), the expected 1019 and 679 bp fragments were detected in the tissues of uninduced LF-GUS plants, respectively (**Figures 6A–C**). No LF-P1/LF-P2 or LF-P3/LF-P4 fragments were detected in the tissues of induced LF-GUS plants except for line #42 showing a faint band, indicating highly efficient DNA recombination in induced plants. By contrast, PCR amplifications using primers LF-P1/LF-P4 generated a 626 bp fragment consistent with reconstitution of the *pUbi-loxP-FRT-gus* transcription unit (**Figure 6D**) in induced LF-GUS plants, except that a faint band was detected in uninduced line # 22 (**Figures 6A–D**), indicating no DNA or almost no DNA recombination in uninduced plants. The PCR products of LF-P1/LF-P4 fragments amplified from induced plants of the four LF-GUS plants were sequenced. And sequencing results from all four lines were identical, showing that the maize ubiquitin-1 promoter was contiguous with one copy of *loxP-FRT* sequence, followed by the *gus* sequence (**Figure 6D**). DNA isolated from LF-GUS-#15 and LF-GUS-#21 plants was double cut with *Xho*I and *Spe*I (**Figure 6D**), and Southern blot analysis was performed using a 3′-end fragment of the maize ubiquitin-1 promoter as a probe. As showed in **Figure 6E**, an expected 4.2 kb band was detected in uninduced LF-GUS-#15 and LF-GUS-#21. By contrast, a 1.4-kb band was detected in induced LF-GUS-#15 and LF-GUS-#21 plants, confirming the recombination events in LF-GUS plants.

### Inducible Silencing of Endogenous *OsPDS* in Transgenic Rice Plants

**FIGURE 7:**
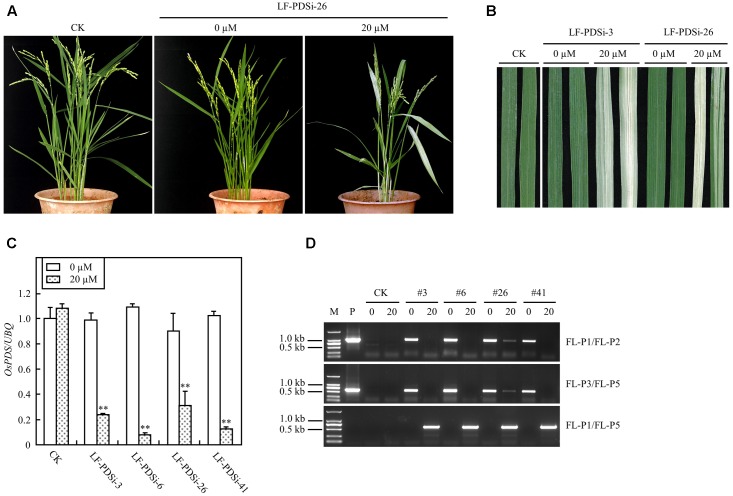
Induced *OsPDS* silencing in mature LF-PDSi transgenic rice plants. **(A)** Phenotype of mature plants developed from seeds soaked in liquid solution with 0 or 20 μM estradiol. CK, non-transgenic wild type control; LF-PDSi-26, pXCLF-PDSi-transformed rice line #26. **(B)** Phenotype of leaves from mature plants developed from seeds soaked in liquid solution with 0 or 20 μM estradiol. CK, non-transgenic wild type control; LF-PDSi-3, LF-PDSi-26, pXCLF-PDSi-transformed rice line #3 and #26. **(C)** qRT-PCR analysis of endogenous *OsPDS* mRNA level in mature plants developed from seeds soaked in liquid solution 0 or 20 μM estradiol. CK, non-transgenic wild type control; LF-PDSi-3, LF-PDSi-6, LF-PDSi-26, LF-PDSi-41, pXCLF-PDSi-transformed rice line #3, #6, #26, and #41. Statistical significances (^∗∗^*P* < 0.01) between plants developed from seeds soaked in 0 or in 20 μM estradiol were analyzed by Student’s *t*-test. **(D)** PCR analysis of chemical-induced site-specific DNA recombination in LF-PDSi transgenic rice plants. P, plasmid control pXCLF-PDSi; CK, non-transgenic wild type control; #3, #6, #26, and #41, pXCLF-PDSi-transformed rice line #3, #6, #26, and #41; 0, plants developed from seeds soaked and germinated in liquid solution with 0 μM estradiol; 20, plants developed from seeds soaked and germinated in liquid solution with 20 μM estradiol.

In addition to ectopic expression of target genes, the XCLF system was also tested for gene knock-down in rice plants. A RNAi construct pXCLF-PDSi (**Figure 1**), which contained an hpRNAi cassette targeted to a rice phytoene desaturase (*OsPDS*) gene, was developed and introduced into rice by *Agrobacterium*-mediated transformation. A total of 14 independent T_0_ plants (named LF-PDSi) were obtained, and no uninduced plants showed detectable albino phenotype. In the T_1_ generation, eight lines of induced transgenic plants showed a clear albino phenotype (**Figures 7A,B**), whereas all transgenic lines developed from seeds geminated in liquid solution without estradiol grew similar to non-transgenic control plants. Real-time RT-PCR analysis showed that the endogenous *OsPDS* mRNA level was greatly reduced in the induced plants (**Figure 7C**). Using the same primers LF-P1, LF-P2, and LF-P3 used for detecting LF-GUS plants, and a specific primer LF-P5 targeted to the hpRNAi cassette of the *OsPDS* fragment (**Figure 1**), PCR analysis detected LF-P1/LF-P2, LF-P3/LF-P5 non-recombinant fragments only in uninduced plants, except for induced line #26 showing faint amplified bands (**Figure 7D**). By contrast, PCR analysis detected a 583 bp LF-P1/LF-P5 recombinant fragment only in induced LF-PDSi plants (**Figure 7D**). Moreover, sequencing results of the LF-P1/LF-P5 amplified products from induced LF-PDSi plants indicated the accurate reconstitution of the recombinant cassette of *pUbi-loxP-FRT-hpRNAi* (*OsPDS*) (Supplementary Figure S2). These results confirmed that the albino phenotype in induced LF-PDSi plants resulted from knock-down of *OsPDS* gene expression mediated by chemically regulated recombination of the XCLF system.

## Discussion

In previous studies, gene induction systems such as the GVG system (Ouwerkerk et al., 2001; Yoshida et al., 2009), the XVE system (Sreekala et al., 2005; Okuzaki et al., 2011), and the GAL4-ER-VP16 system (Zhang et al., 2007) have been applied to conditionally express target genes in rice. GVG consists of a DNA-binding domain of the yeast transcription factor GAL4, a VP16, and a rat glucocorticoid receptor (Aoyama and Chua, 1997). The transcriptional activity of GVG is regulated by glucocorticoid dexamethasone, however, it has been found to cause growth defects in *Arabidopsis* (Kang et al., 1999), lotus (Andersen et al., 2003), tobacco (Amirsadeghi et al., 2007), as well as in rice (Ouwerkerk et al., 2001). The XVE and the GAL4-ER-VP16 systems were developed based on the human estrogen receptor binding domain, and both were regulated by estradiol. While it has not been determined whether the GAL4-ER-VP16 system has toxic or physiological effects on rice plants (Zhang et al., 2007), transgenic rice seedlings expressing a XVE-controlled GFP construct showed no morphological differences compared with non-transgenic control lines (Okuzaki et al., 2011). Similarly, in our study, we observed similar plant heights and seed setting rates between XVE transgenic lines and non-transgenic wild type plants. However, it remains to be investigated whether the XVE system could affect other important agronomic traits in rice.

By using the firefly luciferase system as a reporter, Zhang et al. (2007) demonstrated that the inducibility of target gene expression controlled by the GAL4-ER-VP16 system could reach up to 10,000-fold in rice tissues directly exposed to estradiol. In rice calli stably transformed with a XVE-controlled *gfp* construct, while no GFP signal was detected in calli cultured in media without estradiol, GFP signals were detected in the entire surface of calli cultured in media containing >5 μM estradiol within 2 days (Okuzaki et al., 2011). Similarly, in our study, high inducibility of GUS was detected in *pUX-GUS*-transfected rice protoplast, and in X-GUS calli or X-GUS leaf pieces upon estradiol induction. More importantly, no GUS or almost no GUS expression was observed in uninduced calli or leaf pieces (**Figures 2B,C**), confirming again that the XVE system is highly inducible and tightly regulated in rice cells. However, in intact plantlets induced with estradiol via root immersion, while the roots of the plantlets displayed high inducibility of GUS, the stems and the leaves expressed a much lower level of GUS. Okuzaki et al. (2011) discussed two possible reasons–inefficient estradiol uptake or low XVE expression level, for low level of target gene induction in the leaves of the intact plantlets. Unlike the previous study in which XVE expression was controlled by a weak G10-90 promoter (Okuzaki et al., 2011), in our study, *XVE* was driven by a maize ubiquitin-1 promoter (**Figure 1**), which gives constitutive and high-level expression of transgenes in rice (Christensen and Quail, 1996). In addition, in the present study, estradiol was applied at a high concentration of 20 μM, which was two times higher than the reported saturated concentration for induction (Okuzaki et al., 2011). The fact that GUS activities in the roots of estradiol-induced X-GUS seedlings were about two times higher than that of 35S-GUS plants (**Figure 3B**) also suggested that estradiol uptake and XVE level should be sufficient for activating target gene induction. Therefore, the most likely reason for low inducibility of target gene expression in the leaves of intact plantlets could be that systemic movement of estradiol was limited within the rice plant.

To meet the need for chemical-regulated, high-level expression of target gene in intact rice plants, we have designed an alternative approach by employing the XVE system coupled with a site-specific recombination cassette. In the backbone of the original XVE constructs *pER8* (Zuo et al., 2000), the selectable *hpt* gene for hygromycin resistance was controlled by a weak nopaline synthase promoter (Sanders et al., 1987). In the constructs pXCLF-GUS and pXCLF-PDSi, we replaced the nopaline synthase promoter with a strong CaMV 35S promoter (**Figure 1**). Transformation experiments indicated that this modification greatly enhanced the rice transformation efficiency of the constructs (Supplementary Table S3). By using a GUS reporter, we tested the feasibility of the XCLF system in rice. Histochemical staining of LF-GUS rice seeds soaked in estradiol solution for 3 days showed that GUS was expressed strongly in the germinating embryo (**Figure 4A**), indicating that uptake of estradiol by rice seeds at early stages of germination could efficiently induce Cre/*loxP-FRT*-mediated DNA excision in rice embryos. Consistent with this, rice seedlings and matured plants developed from induced seeds displayed a constitutive GUS expression pattern (**Figures 4B**, **5A–D**), and the GUS expression level in the roots, stems, or leaves were higher or at least similar to that of transgenic rice plants expressing GUS under control of a 35S promoter (**Figures 5B–D**). This property of the XCLF system would allow target genes to be expressed at the desired high level for functional characterization. In the present study, the XCLF system was also tested for knocking down target gene expression. We tested the XCLF system using an hpRNAi cassette for the *OsPDS* gene. The clear albino phenotype and the reduction of *OsPDS* mRNA of induced LF-PDSi plants indicated that the system could be applied in knocking down endogenous gene expression in rice with high efficiency.

In a previous report, Sreekala et al. (2005) observed that DNA recombination mediated by a XVE-controlled Cre/*loxP* system was not efficiently induced in the germline cells in rice. In the present study, we detected highly efficient induced DNA excision in germinating rice embryos. Molecular analyses of induced rice plants displaying strong recombinant phenotypes showed that complete or almost complete DNA excision occurred in the tested plants (**Figures 6A–C**, **7D**). The possible reason for high efficiency of induced DNA recombination in our experiments could be the efficient uptake of estradiol in germinating embryos by the soaking treatment of rice seeds. Unlike previous work, where transgenic seeds were germinated on solid inductive media (Zuo et al., 2001; Guo et al., 2003; Sreekala et al., 2005), we have optimized a seed-soaking protocol for XVE induction. Since uptake of water is the initial event for plant seed germination (Bewley, 1997), soaking seeds in estradiol solution would allow the inducer to access the embryos and induce Cre recombinase at a very earlier stage, resulting in highly efficient DNA recombination.

## Conclusion

**FIGURE 8:**
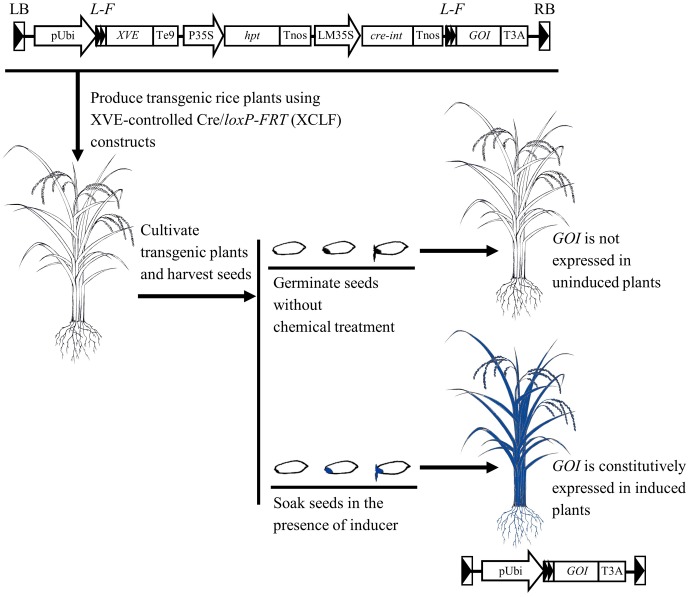
Schematic representation of the chemical-induced, seed-soaking activation procedure for regulated gene expression in rice. In the designed XVE-controlled Cre/*loxP-FRT* (XCLF) constructs, a fragment of expression cassettes of *XVE*, *hpt*, and *cre* genes flanked by two *loxP-FRT* sites in a direct orientation was inserted in between the maize ubiquitin-1 promoter and the *GOI* (gene of interest). In the XCLF-transformed rice plants, *GOI* is not expressed under uninduced condition. Through seed-soaking with estradiol inducer, DNA recombination occurred in embryos would lead to fusion of the maize ubiquitin-1 promoter to the *GOI*, resulting in constitutive activation of *GOI* in intact plants developed from induced seeds.

The chemical-induced, seed-soaking activation procedure based on the XCLF system appears to be effective in regulating gene expression in intact rice plants (**Figure 8**), therefore providing a useful tool for functional genomics and biotechnology applications such as the deletion of selectable marker genes in rice.

## Data Accessibility

The sequence data from this article can be found in the GenBank database, under accession numbers MF434112 (pUX-GUS), MF434111 (pXCL-GUS), MF434113 (pXCLF-GUS), and MF434110 (pXCLF-PDSi).

## Author Contributions

ZJC, G-LW, FW, and SC conceived and designed the experiments. ZJC, QC, CH, XG, ZQC, YL, TH, and MB performed the experiments. ZJC, QC, ZW, FW, and SC analyzed the data. GL and ZW contributed reagents and tools. ZJC and SC wrote the manuscript.

## Conflict of Interest Statement

The authors declare that the research was conducted in the absence of any commercial or financial relationships that could be construed as a potential conflict of interest.

## Footnotes

1 http://www.cambia.org/daisy/cambia/585.html
