# Supplementary material for: A Chemical-Induced, Seed-Soaking Activation Procedure for Regulated Gene Expression in Rice
Source: Front Plant Sci. 2017 Aug 21;8:1447. doi: 10.3389/fpls.2017.01447 (PMC5566991; doi:10.3389/fpls.2017.01447)
Supplement: Supplementary file 1 [file Table_1.DOCX]

**Supplemental Table S 1 Details of Plasmid Construction**

| **Plasmid** | **Details of Plasmid Construction** |
| --- | --- |
| pUX-GUS | The plasmid pUH-GFP2 ( Sreekala et al., 2005) was digested with *Aat*II/*Spe*I to delete the fragment of *C-terminal hpt-LexA-mimi35S promoter-cre-int-Tnos-loxP-gfp*, and a PCR fragment of *Aat*II-*hpt-LexA-mimi35S promoter-Spe*I was amplified using pUH-GFP2 as template with primers UX-F and UX-R (Supplementary Material Table S 1). The PCR fragment was digested with *Aat*II/*Spe*I and then inserted into the *Aat*II/*Spe*I site of pUH-GFP2 to yield the plasmid pUX. A PCR fragment containing a cassette of *Spe*I-*gus-Spe*I was amplified using plasmid pUbiGUS (Chen et al., 2006) as template with primers GUS-F and GUS-R. The fragment was digested with *Spe*I and then inserted into the *Spe*I site in between the *LexA-mimi35S promoter* and the *rbcS 3A* *terminator* of the plasmid pUX. The resulting plasmid was designated pUX-GUS. |
| pXCL-GUS | The plasmid pUH-GFP2 was digested with *Eco*RV/*Spe*I to delete the fragment of *C-terminal cre-int-Tnos-loxP-gfp*, and a PCR fragment of *Eco*RV-*C-terminal cre-int-Tnos-loxP-Spe*I was amplified using pUH-GFP2 as template with primers UH-F and UH-R. The PCR fragment was digested with *Eco*RV/*Spe*I and then inserted into the *Eco*RV/*Spe*I site of pUH-GFP2 to yield the plasmid pUH. The *Spe*I-digested PCR fragment of *gus* was inserted into the *Spe*I site in between the *lox*P recognition sequence and the *rbcS 3A terminator* of the plasmid pUH. The resulting plasmid was designated pXCL-GUS. |
| pXCLF-GUS | The plasmid pUH was digested with *Sma*I/*Aat*II to delete the fragment of *C-terminal XVE-rat glucocorticoid receptor-pea rbcs E9 terminator-nos promoter-small N-terminal hpt*, and a PCR fragment of *Sma*I-*C-terminal XVE-rat glucocorticoid receptor-pea rbcs E9 terminator-Aat*II was amplified using pUH as template with primers NH-F and NH-R. The PCR fragment was digested with *Sma*I/*Aat*II and then inserted into the *Sma*I/*Aat*II site of pUH to yield the plasmid pUXNH. A fragment of *35S promoter*-*small N-terminal hpt* digested with *Bst*XI/*Aat*II from pCAMBIA1300 ([www.cambia.org](http://www.cambia.org)) was inserted into the *Bst*XI/*Aat*II site of pUXNH to yield the plasmid pUXSH.  A PCR fragment of *Apa*I-*C-terminal Maize ubiquitin promoter-loxP-small N-terminal XVE-Mlu*I was amplified using pUXSH as template with primers UL-F and UF-R. The PCR fragment was cloned into the pMD18T vector (Takara, Dalian, China) to yield the plasmid pMD-UL. The plasmid pMD-UL was digested with *Sal*I/*Hin*dIII to delete a *Pst*I site in the backbone of pMD-UL. The re-circularized plasmid of the digested pMD-UL (blunted with Klenow) was digested with *Pst*I/*Mlu*I to delete the fragment of *Pst*I-*loxp-small N-terminal XVE-Mlu*I, and ligated with a *Pst*I/*Mlu*I-digested synthetic fragment of *Pst*I-*loxp+FRT-small N-terminal XVE-Mlu*I (5′-ctgcaggaattcgataaacctaataacttcgtatagcatacattatacgaagttattcaggaagttcctatactttctagagaataggaacttcggaataggaacttcgaattaaatccgggcggaatgaaagcgttaacggccaggcaacaagaggtgtttgatctcatccgtgatcacatcagccagacaggtatgccgccgacgcgt-3′). The resulting plasmid was designated pUXSH-ULF.  To replace the second *loxP* recoginition sequence with *loxP+FRT* fusion sequence in the plasmid pUXSH-ULF, a first-step PCR was performed using pUXSH-ULF as template with primers DLF-F and DLF-R1. By using the DLF-F/DLF-R1 amplicon as template, a PCR fragment of *Sac*II-*C-terminal hpt-Tnos-LexA-mimi35S promoter-cre-int-Tnos-loxp+FRT-Spe*I was amplified with primers DLF-F and DLF-R2. The PCR fragment was digested with *Sac*II/*Spe*I and then inserted into the *Sac*II/*Spe*I site of pUXSH-ULF to yield the plasmid pXCLF. The *Spe*I-digested PCR fragment of *gus* was inserted into the *Spe*I site in between the *loxP+FRT* fusion recognition sequence and the *rbcS 3A terminator* of the plasmid pXCLF. The resulting plasmid was designated pXCLF-GUS. |
| pXCLF-PDSi | To facilitate rapid production of hpRNA constructs, we firstly developed a gateway vector. A gateway-compatible hpRNA cassette digested with *Spe*I from the plasmid pANDA-mini (Miki and Shimamoto 2004) was inserted into the *Spe*I site in between the *loxP+FRT* fusion recognition sequence and the *rbcS 3A terminator* of the plasmid pUXSH-CLF to yield the plasmid pXCLF-GWi. A 470-bp fragment of the rice *OsPDS* gene was amplified using primers PDSi-F and PDSi-R. The PCR fragment was cloned into the Gateway pENTR/D-TOPO cloning vector (Invitrogen, Carlsbad, CA). The resulting plasmid was mixed with pXCLF-GWi, and the LR clonase reaction was carried out to yield the plasmid pXCLF-PDSi according to the manufacturer’s instructions (Invitrogen, Carlsbad, CA). |

**References**

Chen, S., et al. (2006). A highly efficient transient protoplast system for analyzing defence gene expression and protein-protein interactions in rice. *Mol. Plant Pathol.* 7, 417-427.

**Miki, D., and Shimamoto, K. (2004). Simple RNAi vectors for stable and transient suppression of gene function in rice. *Plant Cell Physiol.* 45, 490**-**495.**

**Sreekala, C., et al. (2005). Excision of a selectable marker in transgenic rice (*Oryza sativa* L.) using a chemically regulated Cre/*loxP* system. *Plant Cell Rep.* 24, 86**-**94.**
