# Supplementary material for: A Chemical-Induced, Seed-Soaking Activation Procedure for Regulated Gene Expression in Rice
Source: Front Plant Sci. 2017 Aug 21;8:1447. doi: 10.3389/fpls.2017.01447 (PMC5566991; doi:10.3389/fpls.2017.01447)
Supplement: Supplementary file 2 [file Table_2.DOC]

**Supplemental Table S 2 List of primers used in this study**

| Primers | Sequence: 5ʹ-3ʹ | Comments |
| --- | --- | --- |
| UX-F | GACGTCTGTCGAGAAGTTTCTGATC | Forward primer to amplify the fragment of *Aat*II*-hpt-LexA-mini35S promoter-Spe*I for making pUX-GUS |
| UX-R | ACTAGTGATTGATATCGACTAGCTTCAGCGTGTC | Reverse primer to amplify the fragment of *Aat*II*-hpt-LexA-mini35S promoter-Spe*I for making pUX-GUS |
| GUS-F | gactagtatgttacgtcctgtag | Forward primer to amplify the *gus* gene for making pUX-GUS or other plant expression vectors containing *gus* |
| GUS-R | gactagtcattgtttgcctccctg | Reverse primer to amplify the *gus* gene for making pUX-GUS or other plant expression vectors containing *gus* |
| UH-F | ACTGATATCTCACGTACTGACGG | Forward primer to amplify the fragment of *Eco*RV*-C-terminal cre-int-Tnos-loxP-Spe*I for making pXCL-GUS |
| UH-R | AGTACTAGTGAAGATCTATAACTTCG | Reverse primer to amplify the fragment of *Eco*RV*-C-terminal cre-int-Tnos-loxP-Spe*I for making pXCL-GUS |
| NH-F | ATTCCCCGGGTCCGGGATTTAC | Forward primer to amplify the fragment of *Sma*I*-C-terminal XVE-rat glucocorticoid receptor-pea rbcs E9 terminator-Aat*II for making pXCLF-GUS |
| NH-R | GACGTCCCAACATGGTGGTCAGTTTC | Reverse primer to amplify the fragment of *Sma*I*-C-terminal XVE-rat glucocorticoid receptor-pea rbcs E9 terminator-Aat*II for making pXCLF-GUS |
| UL-F | tgggcccggtagttctacttctgt | Forward primer to amplify the fragment of *Apa*I*-C-terminal Maize ubiquitin promoter-loxP-small N-terminal XVE-Mlu*I for making pXCLF-GUS |
| UL-R | tacgcgtcggcggcatacctgtct | Reverse primer to amplify the fragment of *Apa*I*-C-terminal Maize ubiquitin promoter-loxP-small N-terminal XVE-Mlu*I for making pXCLF-GUS |
| DLF-F | tccgcggctccgggcgtatatgc | Forward primer to amplify the fragment of *Sac*II*-C-terminal hpt-Tnos-LexA-mimi35S promoter-cre-int-Tnos-loxp+FRT-Spe*I for making pXCLF-GUS |
| DLF-R1 | ttcctattctctagaaagtataggaacttcctgaataacttcgta | Reverse primer 1 to amplify the fragment of *Sac*II*-C-terminal hpt-Tnos-LexA-mimi35S promoter-cre-int-Tnos-loxp+FRT-Spe*I for making pXCLF-GUS |
| DLF-R2 | tactagtgaagatctgaagttcctattccgaagttcctattctctaga | Reverse primer 2 to amplify the fragment of *Sac*II*-C-terminal hpt-Tnos-LexA-mimi35S promoter-cre-int-Tnos-loxp+FRT-Spe*I for making pXCLF-GUS |
| PDSi-F | CACCTTATGCGGACATGTCAG | Forward primer to amplify *OsPDS* fragment for making pXCLF-PDSi |
| PDSi-R | CCATTGGGAATAGTCCTGACTAC | Reverse primer to amplify *OsPDS* fragment for making pXCLF-PDSi |
| REB4-F | GGAATGAGTAGCCCGGAACC | Forward primer for real-time qPCR of rice endogenous *REB4* gene |
| REB4-R | AAGAGTGCTCCTGGATTGCC | Reverse primer for real-time qPCR of rice endogenous *REB4* gene |
| Hpt-F | ATGTCCTGCGGGTAAATAGC | Forward primer for real-time qPCR of *Hpt* gene |
| Hpt-R | CCAATGTCAAGCACTTCCG | Reverse primer for real-time qPCR of *Hpt* gene |
| LF-P1 | ggttgggcggtcgttcattcgttc | Primer 1 to detect molecular recombination events in pXCLF-GUS or pXCLF-PDSi transgenic plants |
| LF-P2 | ctcgtcaattccaagggcatcggt | Primer 2 to detect molecular recombination events in pXCLF-GUS or pXCLF-PDSi transgenic plants |
| LF-P3 | gatagtgaaacaggggcaatgg | Primer 3 to detect molecular recombination events in pXCLF-GUS or pXCLF-PDSi transgenic plants |
| LF-P4 | CAACGCTGATCAATTCCACAGTTTTCG | Primer 4 to detect molecular recombination events in pXCLF-GUS transgenic plants |
| LF-P5 | GCGCCGACCCAGCTTTCTTGTAC | Primer 5 to detect molecular recombination events in pXCLF-PDSi transgenic plants |
| qRT-PDS-F | CTTCGCAAGTAGCAGCATC | Forward primer for real-time qRT-PCR of *OsPDS* |
| qRT-PDS-R | CTGTAGAGCACCGAGCCT | Reverse primer for real-time qRT-PCR of *OsPDS* |
| qRT-UB-F | AACCAGCTGAGGCCCAAGA | Forward primer for real-time qRT-PCR of rice internal control *Ubiquitin* gene |
| qRT-UB-R | ACGATTGATTTAACCAGTCCATGA | Reverse primer for real-time qRT-PCR of rice internal control *Ubiquitin* gene |
