# Supplementary material for: A Chemical-Induced, Seed-Soaking Activation Procedure for Regulated Gene Expression in Rice
Source: Front Plant Sci. 2017 Aug 21;8:1447. doi: 10.3389/fpls.2017.01447 (PMC5566991; doi:10.3389/fpls.2017.01447)
Supplement: Supplementary file 3 [file Table_3.DOC]

**Supplementary Table S 3 Comparison of transformation efficiency of constructs pXCL-GUS and pXCLF-GUS**

| Construct | No. of calli  for transformation | No. of hygromycin-  resistant calli | No. of regenerated  transgenic plants | Transformation  efficiency (%) |
| --- | --- | --- | --- | --- |
| pXCL-GUS | 990 | 26 | 2 | 0.20 |
| pXCLF-GUS | 590 | 23 | 18 | 3.05 |
