# Supplementary figures and images for: A Chemical-Induced, Seed-Soaking Activation Procedure for Regulated Gene Expression in Rice
Source: Front Plant Sci. 2017 Aug 21;8:1447. doi: 10.3389/fpls.2017.01447 (PMC5566991; doi:10.3389/fpls.2017.01447)

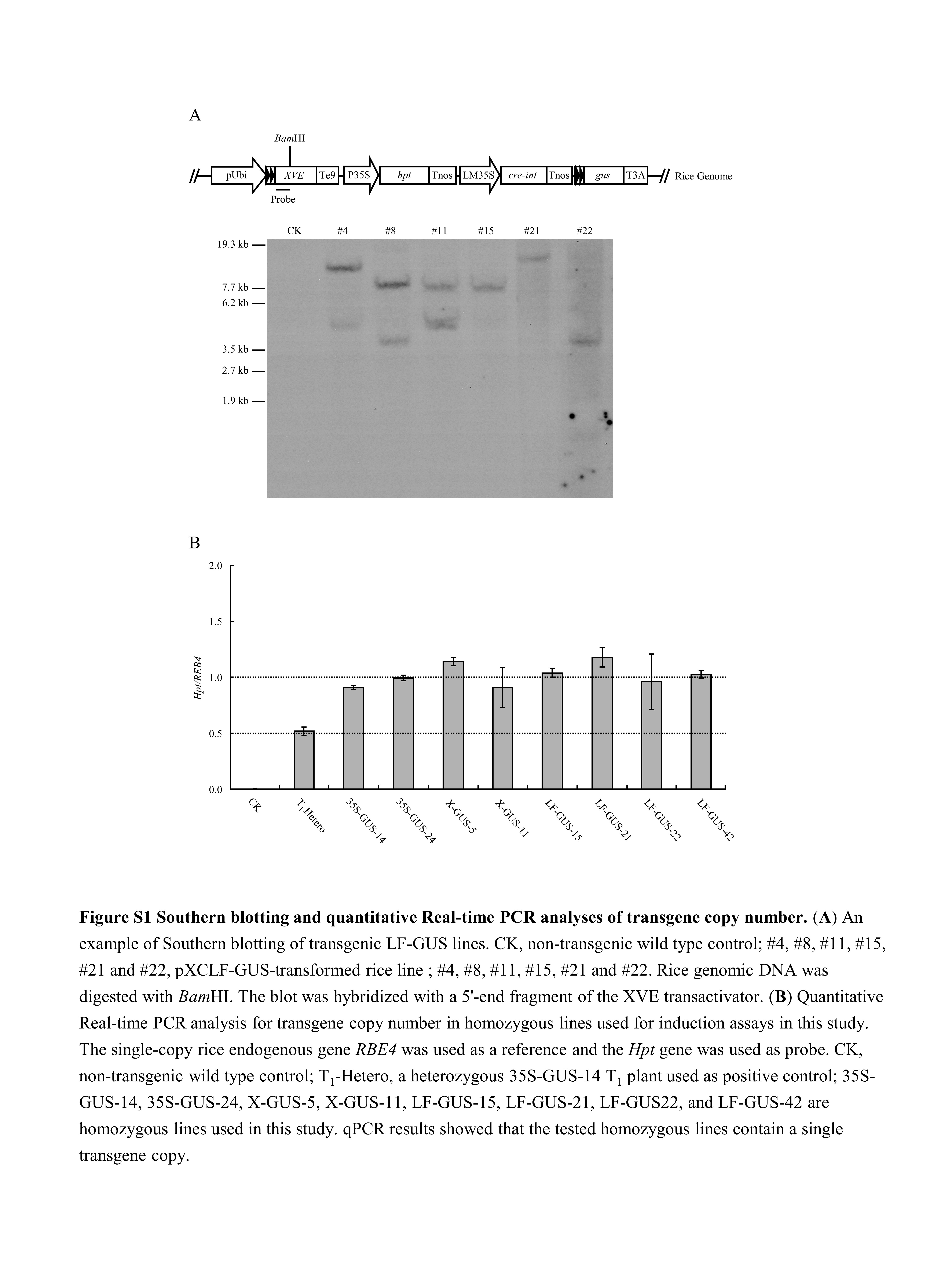

Supplement: Supplementary file 4 [file Image_1.JPEG]

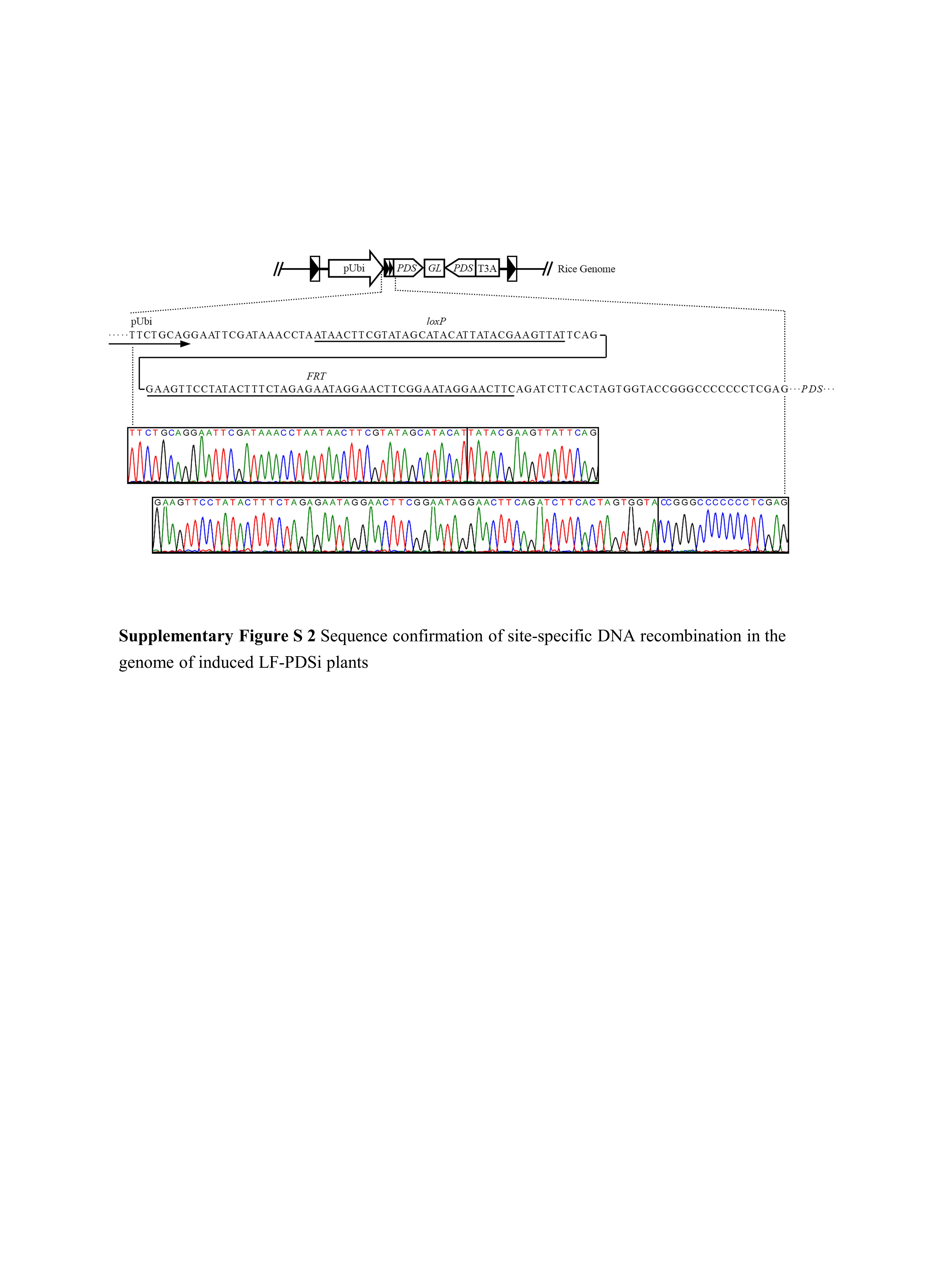

Supplement: Supplementary file 5 [file Image_2.JPEG]
